# Comparison between Track Technique and Conventional Approach for Measuring Artificial Chordae in the Treatment of Anterior Leaflet Prolapse and Flail during Mitral Valve Repair

**DOI:** 10.31083/j.rcm2410301

**Published:** 2023-10-20

**Authors:** Giuseppe Nasso, Giuseppe Santarpino, Raffaele Bonifazi, Flavio Fiore, Gaetano Contegiacomo, Felice Eugenio Agrò, Ignazio Condello, Giacomo Dimita, Francesco Bartolomucci, Khalil Fattouch, Marco Moscarelli, Nicola Di Bari, Giuseppe Speziale

**Affiliations:** ^1^Department of Cardiac Surgery, Anthea Hospital, GVM Care & Research, 70124 Bari, Italy; ^2^Department of Cardiac Surgery, Città di Lecce Hospital, GVM Care & Research, 73100 Lecce, Italy; ^3^Department of Experimental and Clinical Medicine, “Magna Graecia” University, 88100 Catanzaro, Italy; ^4^Department of Anesthesiology, Campus Bio-Medico University Hospital of Rome, 00128 Roma, Italy; ^5^Department of Cardiology, Azienda Sanitaria Locale - BAT, 76123 Andria, Italy; ^6^Department of Cardiac Surgery, Maria Eleonora Hospital, GVM Care & Research, 90135 Palermo, Italy; ^7^Department of Cardiac Surgery, San Carlo di Nancy Hospital, GVM Care & Research, 00165 Rome, Italy

**Keywords:** mitral valve reconstruction, artificial chordae tendinea, height measurement, mitral valve regurgitation

## Abstract

**Background:**

Measuring the chordae tendineae for mitral valve reconstruction is feasible with various techniques. However, the effect of different strategies on the durability of plastics at follow-up is unknown. The study aims to compare a conventional surgical technique for measuring artificial chordae length with our new approach, defined “track technique”.

**Methods:**

We compared the results of patients with anterior leaflet prolapse/flail who underwent mitral valve reconstruction by implanting artificial chordae from January 2020 to January 2022; 22 patients were operated on with a conventional technique, and 25 with our new alternative, “track technique”. Clinical and transesophageal echocardiography data were collected postoperatively and at 2 years of follow-up. The primary outcome was freedom from mitral regurgitation. Secondary outcomes were presentation with New York Heart Association (NYHA) class <2 and leaflet coaptation length ≥10 mm.

**Results:**

The patients of the 2 groups had comparable preoperative risk factors regarding the LogEuroSCORE (*p* = 0.33). Moreover, no difference was observed in terms of the mechanism of mitral valve insufficiency. No hospital or follow-up deaths were recorded for either group. At discharge, no echocardiographic differences were observed in the regarding degree of residual mitral regurgitation, but the measurement of coaptation length was in favor of the alternative group (8.6 ± 1.8 vs. 11 ± 1.4; *p* = 0.04). At 2 years of follow-up (25 ± 9; range 13–37), the NYHA class was not different; however, the number of patients with 1–2+ recurrent mitral regurgitation was significantly higher in the conventional group (8 vs. 4 patients; *p* = 0.02), and the coaptation length was in favor of the alternative group (8.8 ± 1.7 vs. 11 ± 1.7; *p* = 0.04).

**Conclusions:**

We devised both techniques to prove effective in achieving good valvular continence, but a significantly greater coaptation length was obtained with our track technique at the 2 years follow-up.

## 1. Introduction

Mitral valve repair (MVr) is regarded as the optimal surgical approach for treating degenerative mitral regurgitation (MR) [1, 2]. Althrough Carpentier initially popularized resection repair techniques for MVr [3], non-resectional procedures, such as chordal replacement with expanded polytetrafluoroethylene, have become more commonly utilized, particularly in cases of anterior prolapse [4, 5, 6, 7] to preserve the subvalvular apparatus. Accurately adjusting the length of the artificial chordae is a crucial and challenging step in this approach. Incorrect measurement of chordal length can result in repair failure and early recurrence of MR [8, 9]. Our initial experience (conventional technique) with a straightforward and reproducible technique for precisely adjusting the length of artificial chordae during MVr in patients with anterior leaflet disease has already been published [10]. However, the effectiveness in terms of the durability of the track technique, compared with conventional measurements of artificial chordae, has not been evaluated to date.

## 2. Materials and Methods

Retrospective data collection was conducted between January 2020 and January 2022, encompassing the computerized medical records of patients who underwent MVr at a single cardiac surgery center. The data specifically focused on patients with anterior leaflet prolapse or flail.

All surgical procedures were conducted using general anesthesia and single- or double-lumen orotracheal intubation to selectively exclude the right lung. In cases of isolated MR, the preferred approach was a right anterior mini-thoracotomy. In contrast, patients with concurrent coronary artery or valve disease underwent standard median sternotomy. During the operation surgeries, cardiopulmonary bypass was performed, employing double venous cannulation and bicaval snaring, with mild hypothermia maintained between 34 °C and 36 °C. The priming solution for the bypass circuit included 1250 mL of Ringer’s crystalloid acetate solution and the Stöckert S5 heart-lung machine as the perfusion system. A closed circuit was employed for myocardial protection using cold antegrade blood cardioplegia with a heat exchanger. Additionally, an infusion syringe pump in series and Saint Thomas solution with procaine were used, with cardioplegia administration repeated every 30 minutes. Guiraudon’s biatrial techniques were utilized for patients undergoing surgery through a median sternotomy. At the same time, a left atriotomy approach was employed for patients treated via a right mini-thoracotomy to access the mitral valve.

The severity of MR was assessed and graded based on the 2017 European Society of Cardiology Guidelines for the Management of Valvular Heart Disease [11]. We divided the population into 2 groups. The first comprised patients who underwent conventional chordae measurements at the Anthea Hospital Cardiac Surgery Center of the Villa Maria Group (GVM) in Bari. In the conventional technique, the suture was first tied to the fibrous tip of the papillary muscle (PM), and the 2 ends were fixed to the free edge of the prolapsing anterior leaflet in a V-shape. Next, a new chord length was measured by bringing the free edge of the valve to the level of the anterior annulus. The length could also be compared to healthy non-elongated native chords in the adjacent area. Then both ends of the sutures would be passed again through the free edge and tied on the ventricular side of the leaflet to prevent the knot from interfering with the coaptation zone [12].

**Fig. 1.**
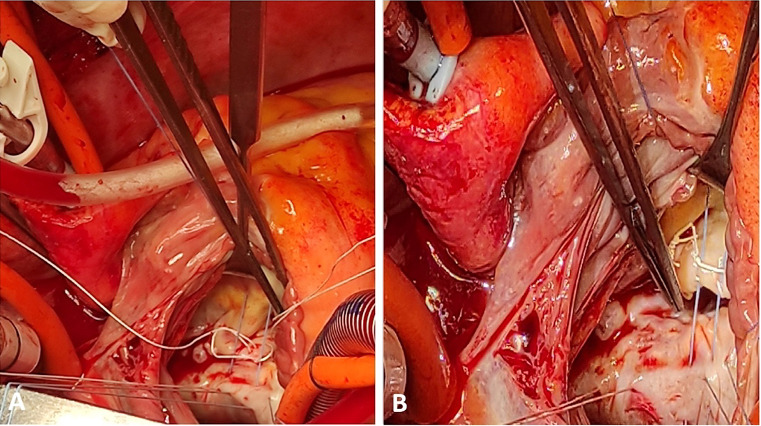
**“Track technique: steps I-II-III”.** (A) To target the specific area affected by the diseased leaflet segment, a single Ethibond suture is threaded through the anterior annulus. This precise placement of the suture allows for targeted and effective treatment of the affected region during the mitral valve repair procedure. To create a temporary guide for chordal attachment, the needle tips of the Ethibond suture are carefully inserted through the 2 loops of the previously implanted artificial neo-chord. Afterward, they are threaded through the posterior annulus of the opposing segment. Finally, the suture ends are tied separately, forming a temporary guide that aids in securing the neo-chords in their proper position. This technique ensures accurate alignment and attachment of the neo-chords, facilitating effective mitral valve repair. (B) The 2 ends of each neo-chord are adjusted to the height of the guide and tied right above it.

**Fig. 2.**
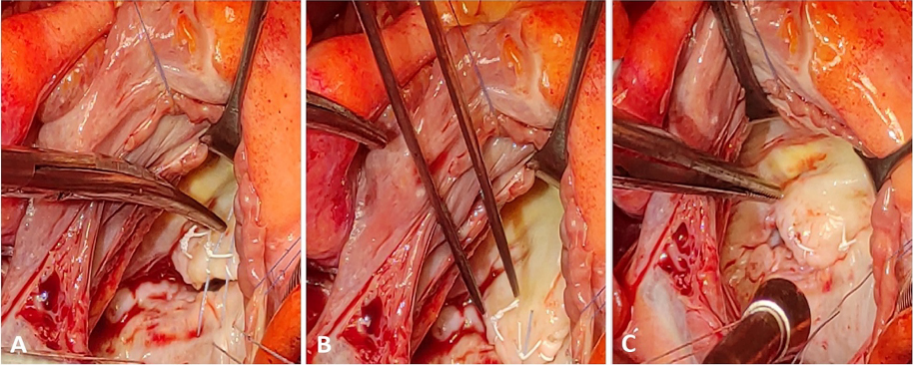
**“Track technique: steps IV-V”.** (A) Once the final adjustment of the neo-chord has been made, the annular Ethibond suture is cut to separate it from the repair site. (B) The guide that was used during the procedure is carefully removed from the surgical area. (C) The mitral valve repair procedure at its conclusion, ensuring the restoration of proper valve function.

The second group, operated in the same center, underwent our original technique for the chordae measurement, and results have been previously published [10]. In summary, our technique involves the following 5 key steps: *Step I*: Once the prolapsing segment and elongated or ruptured chordae of the anterior leaflet are identified, the surgeon threads one or more 4-0 expanded polytetrafluoroethylene (ePTFE) chordae through the fibrous head of the anterior papillary muscle. To prevent any damage before and after the suture to the papillary fibrous portion, a precautionary measure is taken by utilizing 2 pledgets to ensure the integrity and protection of the tissue. *Step II*: The ePTFE suture is threaded twice through the prolapsing anterior leaflet, moving from the ventricular to the atrial side. This process is done at a distance of approximately 5 mm, resulting in the creation of 2 loops. It is important to note that these loops are left untied at this stage. *Step III*: To create a temporary guide for chordal attachment, a single Ethibond suture is threaded through the anterior annulus at a specific location corresponding to the diseased leaflet segment. The needle tips of the Ethibond suture are then individually passed through the 2 loops of the previously implanted artificial neo-chord. Subsequently, they are threaded through the posterior annulus of the opposing segment, maintaining a distance of a few millimeters between them. Finally, the suture is tied to secure the chordal attachment (Fig. 1A,B). During this process, utmost care is taken to prevent injury to the surrounding structures, such as the circumflex artery and aortic valve. Special attention is given to the anchoring of the Ethibond suture to the anterior and posterior annulus, ensuring the safety of the nearby anatomical components. *Step IV*: The 2 free ends of each neo-chord are subsequently adjusted to align with the height of the annular Ethibond suture. They are then tied securely just above the Ethibond suture. This adjustment and tying process ensures proper positioning and tensioning of the neo-chords in relation to the annulus, facilitating effective mitral valve repair. *Step V*: A thorough inspection of the neo-chord is conducted before removing the guide. Necessary adjustments were made at this stage to ensure optimal placement and tension. Once the inspection and adjustments were complete, the annular Ethibond suture was cut, and the guide was carefully removed from the surgical site (Fig. 2A–C).

In both groups, the MVr procedure was finalized by treating any accompanying lesions on the posterior leaflet using resection techniques. Additionally, to ensure a proper MVr approach, a complete MEMO 3DTM (Corcym, England) was implanted in all cases.

The study protocol was approved by our Institutional Review Board. Since all the patient data were treated anonymously, given the study’s retrospective nature, and no additional diagnostic or therapeutic procedures were conducted on patients, individual informed consent was not deemed necessary. Pre, intra, and postoperative outcomes were compared between the 2 groups. All patients were contacted for a follow-up at 2 years.

### Statistical Analysis

The data were analyzed using SPSS (Statistical Package for Social Sciences, SPSS Inc, Chicago, IL, USA) software version 11.0 for Windows. Patients were divided into 2 groups: conventional and alternative, with the conventional approach and track technique, respectively, to measure chordae tendineae. Continuous variables were presented as mean ± S.D., and categorical variables as percentages. Group comparisons were made using the χ^2^ test for categorical variables and 1-way ANOVA for continuous variables. Student *t*-test was used for continuous variables. Postoperative complications were defined as those occurring within 30 days of surgery; therefore, no time-to-event analysis was used to identify risk factors for postoperative complications. The follow-up was performed by clinical and echocardiographic evaluation.

## 3. Results

The preoperative characteristics of the 2 groups are described in Table 1, which shows no significant differences between the 2 groups in the recorded variables, such as risk factors and the anatomical-echocardiographic characteristics of the mitral valves to be repaired. Table 2 shows the intraoperative data, which also did not differ between the 2 groups, either in terms of the duration of the procedures or the number of chordae used. We emphasize that all patients who underwent these repair procedures involving the anterior mitral leaflet had satisfactory functional and echocardiographic results, and no patient required valve replacement.

**Table 1.** **Preoperative characteristics**.

Variable	Conventional	Alternative	*p*-value
	n = 22	n = 25	
Mean age	68 ± 2	68.5 ± 4.2	0.77
Sex, female	6 (27)	9 (36)	0.09
Concomitant surgery	5 (23)	5 (20)	0.42
Active smoke	6 (27)	6 (24)	0.33
COPD	3 (14)	3 (12)	0.65
Atrial fibrillation	9 (41)	9 (36)	0.11
Diabetes mellitus	11 (50)	11 (44)	0.12
Dyslipidemia	13 (59)	13 (52)	0.17
Systemic hypertension	20 (91)	20 (80)	0.09
Chronic renal insufficiency	7 (32)	6 (24)	0.07
Isolated AML prolapse	15 (68)	15 (60)	0.12
Bi-leaflets prolapse	7 (32)	10 (40)	0.06
Length of the anterior leaflet (mm)	32 ± 4	30 ± 5	0.25
Length of the posterior leaflet (mm)	15 ± 5	16 ± 4	0.28
Antero‐posterior diameter of annulus	46 ± 4	47 ± 7	0.18
LVEF	43 ± 7	45 ± 10	0.31
Log EuroSCORE	6 ± 1	5.7 ± 2.4	0.33

Abbreviations: AML, anterior mitral leaflet; COPD, chronic obstructive pulmonary disease; LVEF, left ventricular ejection fraction.

**Table 2.** **Intraoperative Characteristics**.

Variable	Conventional	Alternative	*p*-value
	n = 22	n = 25	
Mini right thoracotomy	17 (77)	20 (80)	0.15
Aortic cross-clamp time, min	60 ± 5.9	61 ± 11	0.66
Cardiopulmonary bypass time, min	93 ± 8.5	95 ± 16	0.59
Ring size	34 (32–38)	34 (32–38)	-
Number of chordae	2 (1–4)	2 (1–4)	-
Concomitant AF surgery	6 (27)	6 (24)	0.43

Abbreviations: AF, atrial fibrillation.

**Fig. 3.**
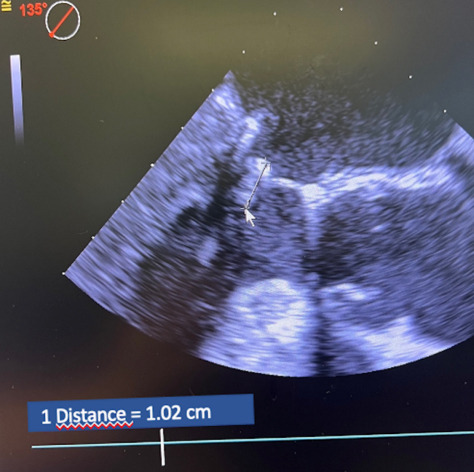
**At the 2-year echocardiographic follow-up using the mid-esophageal long axis view, the coaptation length (see white arrow) was measured to be 1.02**.

**Fig. 4.**
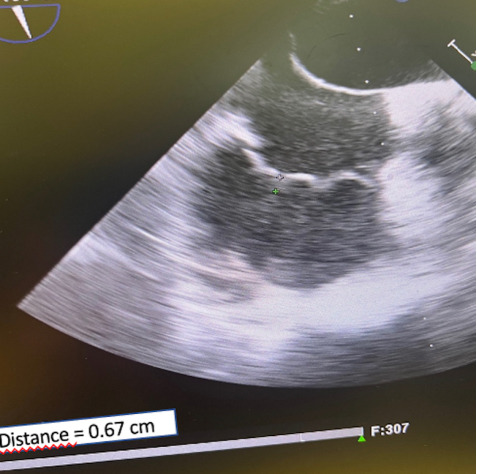
**Conventional technique.** At the 2-year follow-up, echocardiographic control (mid-esophageal long axis coaptation length was 0.67.

The postoperative results, shown in Table 3, recorded comparable data in terms of hospital clinical outcomes. In terms of echocardiography, although both groups have over 95% of patients discharged in the absence of residual mitral regurgitation and the remaining patients with a trivial/1+ grade, patients treated with chordae measurement with our original technique recorded a significantly higher coaptation length than that obtained by measuring chordae with the conventional technique (see Table 3). At the 2-year follow-up (25 ± 9; range 13–37), all the patients reached as no deaths were recorded at the follow-up in both groups, and no cases of procedure failure requiring reoperation or endocarditis were observed. Furthermore, the NYHA functional class at 2 years was not different between the 2 groups (conventional: 1.2 ± 0.4; alternative: 1.1 ± 0.3; *p* = 0.32), while the number of patients with 1+ or 2+ mitral regurgitation was significantly higher in the group of conventional patients (conventional: 8 patients (36%); alternative: 4 patients (16%); *p* = 0.02), and the coaptation length was in favor of the alternative group (8.8 ± 1.7 vs. 11 ± 1.7; *p* = 0.04) (Table 4 and Figs. 3,4).

**Table 3.** **Postoperative results**.

Variable	Conventional	Alternative	*p*-value
	n = 22	n = 25	
30-day mortality, No. (%)	0	0	-
ICU length of stay, d	2.9 ± 0.8	3 ± 1	0.11
Hospital stay, d	8.8 ± 1.5	9 ± 2	0.33
Mitral insufficiency post-op	0 in 20 (95.5)	0 in 23 (96)	0.09
	1 in 2 (4.5)	1 in 2 (4)	
Atrial fibrillation	6 (27)	5 (20)	0.08
Ventilation >24 h	1 (4.5)	1 (4)	0.15
Coaptation length (mm)	8.6 ± 1.8	11 ± 1.4	0.04

Abbreviations: ICU, intensive care unit.

**Table 4.** **Follow-up results**.

Variable	Conventional	Alternative	*p*-value
	n = 22	n = 25	
Reoperation	0	0	-
Endocarditis	0	0	
NYHA	1.2 ± 0.4	1.1 ± 0.3	0.32
Mitral regurgitation 1+/2+, No. (%)	8 (36%)	4 (16%)	0.02
Mortality	0	0	0.11
Coaptation length (mm)	8.8 ± 1.7	11 ± 1.7	0.04

Abbreviations: NYHA, New York Heart Association.

## 4. Discussion

Artificial chordae play a crucial role in MVr by enhancing leaflet coaptation and allowing the use of larger annuloplasty rings, offering advantages over resection techniques [13]. However, precise measurement of the artificial chordae length is essential to achieve optimal mitral valve function and long-lasting outcomes. The most commonly used method for chordal sizing involves inflating the left ventricle with saline to achieve leaflet apposition. Once this is achieved, a standard annuloplasty ring is implanted, and the neo-chordal sutures are secured [6, 14, 15, 16]. Over time, various other techniques have been published for assessing chordal length [17, 18, 19, 20, 21, 22, 23, 24, 25, 26], expanding the options available for surgical planning and execution.

In our conventional technique, the initial step involved tying the suture to the fibrous tip of the papillary muscle (PM), with the 2 ends then secured to the free edge of the prolapsing anterior leaflet in a V-shaped configuration. The measurement of a new chord length was obtained by aligning the free edge of the valve with the level of the anterior annulus. This approach allowed for precisely determining the appropriate length for the artificial chordae [12].

Alternatively, our track technique for achieving the correct length of artificial chordae is easy to implement. It involves creating a temporary guide for neo-chordal attachment between the anterior and posterior annulus, specifically targeting the segment affected by the disease. Unlike other structures within the mitral valve apparatus, the annulus provides stability during cardioplegic arrest, making it a reliable intracardiac landmark for guide placement.

The advantage of our approach is its simplicity and efficiency, as it eliminates the need for preoperative echocardiographic or intraoperative measurements. The track technique saves time and simplifies the surgical process. The repair was successfully completed using a complete MEMO 3DTM, with a median ring size of 34 (32–38) mm, ensuring a comprehensive treatment for mitral valve pathology. Our approach has already been shown to be effective, with good follow-up results [10]. However, more case studies would be needed to confirm our results which still carry the limitation of being a single surgeon experience. On the other hand, our approach had not yet been compared to that of other artificial chordae measurement techniques, nor its ability to make the effect lasting in relation to its ability to result in a coaptation length greater than 10 mm has been studied.

The association between a coaptation length greater than 10 mm and a greater durability of the plastic is already known in the literature [27, 28, 29]. We interpreted this result by concluding that it is not the repair technique that influences the long-term result of the mitral valve reconstruction but the coaptation length. Indeed, achieving the correct length of the artificial chordae and selecting the appropriate ring size based on the measurement of the anterior leaflet area and the anteroposterior diameter of the mitral annulus are crucial factors in determining the optimal coaptation length. These measurements are essential for ensuring proper leaflet apposition and mitral valve competence. By accurately determining the appropriate chordal length and selecting the right ring size, surgeons can achieve an effective repair that promotes optimal valve function and helps prevent regurgitation. In other words, our patients could also be subjected to another technique with measurement of the chordae and not to our original technique, obtaining the same result if the coaptation length was still greater than 10 mm. Our conclusion is, therefore, that given that the 2 techniques were performed by the same surgeon in patients with similar anatomical features, our technique is no longer effective per se in reducing the risk of relapse of mitral regurgitation but facilitates the achievement of a greater coaptation between the two mitral leaflets.

The limitation of our study consists of the need for a broader case study and the reproducibility analysis by having other surgeons perform this technique. Finally, it needs to be seen whether by further extending the follow-up to more than 2 years, the gap between conventional patients and patients treated with our technique widens further or remains stable for the results recorded at 2 years.

## 5. Conclusions

In conclusion, all mitral repair techniques with implantation of artificial chordae on the anterior mitral leaflet require standardization to measure the chord itself correctly. Our technique does not prove to be more effective than another technique that we have called “conventional” but has made it possible to obtain an equally competent valve but with a greater coaptation length. This anatomical finding was associated with a result of mitral valve repair effectiveness at the best follow-up, awaiting results at even greater time intervals and evaluation of the technique if performed by other surgeons experienced in mitral valve repair with cords measured with other systems.

## Data Availability

The datasets used and/or analyzed during the current study are available from the corresponding author on reasonable request.
